# Cancer screening in patients with acromegaly: a plea for a personalized approach and international registries

**DOI:** 10.1007/s11154-025-09957-6

**Published:** 2025-03-15

**Authors:** Luigi Demarchis, Sabrina Chiloiro, Antonella Giampietro, Laura De Marinis, Antonio Bianchi, Maria Fleseriu, Alfredo Pontecorvi

**Affiliations:** 1 Dipartimento Di Medicina Traslazionale, Università Cattolica del Sacro Cuore, Rome, Italy; 2 Dipartimento Di Medicina Interna, Endocrinologia E Diabetologia, Fondazione Policlinico Universitario A. Gemelli IRCCS, Università Cattolica del Sacro Cuore, Rome, Italy; 3 Pituitary Center, and Departments of Medicine, and Neurological Surgery, Oregon Health & Science University, Portland, OR USA

**Keywords:** Somatotropinomas, Neoplasms, Screening, Colon, Breast, Prostate, Thyroid

## Abstract

Acromegaly is a rare condition, and often diagnosis is delayed by several years, for most patients. Acromegaly is characterized by short and long-term respiratory, cardiovascular and metabolic comorbidities, with possible impact on mortality. In the last two decades, life expectancy has progressively increased in part due to a reduction in biochemically active disease, multidisciplinary treatment approaches and a reduction in complications, and the availability of new drugs. Of note, a leading cause of mortality, cardiovascular comorbidity, has been replaced by cancer(s). As such, neoplasms more frequently observed (colon, thyroid, breast, prostate, and stomach) in patients with acromegaly are receiving increased attention. Chronic exposure to increased growth hormone serum levels may contribute to an increase in the occurrence and progression of cancers. Various efforts have been made to determine the pathogenetic mechanisms involved. However, there are no clear medical-related societal agreement(s) in relation to screening methods or timing regarding neoplasm(s) diagnosis in patients with acromegaly. Additionally, independent and dependent risk factor data in patients with acromegaly is lacking. International/national registries could help lay the groundwork to better study the impact of cancer(s) in patients with acromegaly and subsequently lead to and validate the most appropriate diagnostic and therapeutic path forward.

## Background

Acromegaly is a systemic disease, caused by the excessive secretion of the growth hormone (GH), due in most cases to the presence of a GH-secreting pituitary adenoma [1]. Overall, acromegaly is a rare disease, with a slightly increased prevalence and incidence over time [2]. While, in all countries, epidemiology is not well-defined disease prevalence ranges from 28 to 137 cases per million with an incidence of 2–11 cases per million/year [3–7].

The mortality rate in patients with acromegaly is increased in those who have active disease, as compared to those who are living with controlled acromegaly and the general population. Standardized mortality ratio (SMR) is reportedly 0.98 (studies that enrolled patients on adjuvant treatment with somatostatin receptor ligands; SRLs) and 2.11 (studies with patients undergoing only surgery and/or radiation therapy) [8].

Acromegaly-related mortality is directly correlated with disease activity [9], and is historically associated with cardiovascular, respiratory, and metabolic comorbidities [10]. With improvements in cardiovascular comorbidity management, an increase in the occurrence of neoplasias has been noted. In a study by Ritvonen et al. in 2016, deaths in patients with acromegaly after diagnosis, from cardiovascular vs neoplastic causes were more frequent (44% vs 28%) in the first 10 years, while deaths from neoplastic vs cardiovascular causes were more frequent (35% vs 23%) in following 20 years [11].

Treatment goal(s) are multifactorial; however disease *remission* is key. Remission defined as normal age-adjusted insulin-like growth factor-I (IGF-I) levels, postoperatively (at 12 weeks after surgery) and in patients undergoing medical therapy; notably measured in the last week before the next administration for injectable SRL therapy [12]. Multimodal treatment (surgery and/or medical therapies) [13], has led to increased numbers of patients with controlled disease and resulted in life expectancy increases; with reduced disease mortality rate(s) and decreased frequency of comorbidities [14]. Therefore, cancer screening has become of increasing importance in the management of patients with acromegaly [15–17].

Although the underlying pathogenetic mechanisms are not yet fully understood, increased serum IGF-1 and insulin-like growth factor binding protein-3 (IGF-BP3) levels remain correlated with an increased risk of malignancy development [18]. There are several mechanisms by which GH and IGF-1 may promote cell differentiation that result in neoplasms. Increased serum GH and IGF-1 levels promote several pro-tumor mechanisms including increased tissue proliferation via signal transducer and activator of transcription 5 (STAT5) pathways, activation of mitogen-activated protein kinase (MAPK), and increased metabolic activity of phosphatidylinositol 3-kinase/protein kinase-B (PI3K/AKT) [19]. Many mechanisms and risk factors that contribute to an increased likelihood of developing neoplasms in patients with acromegaly remain, however, to be elucidated.

A prospective study in 2024, conducted in a large cohort of patients with acromegaly reported that cancer risk correlated with both the degree and duration of excess IGF-1 [20].

Over the past two decades, various scientific societies, using national registries [21–23]have analyzed cancer risks and need for screening. However, an association between acromegaly and second neoplasms as well as the underlying mechanisms of carcinogenesis have not yet been fully elucidated.

Low GH levels have been correlated with a reduced incidence of neoplasia, though it is still controversial whether GH plays a direct role in promoting per se the occurrence of neoplasia; the possible correlation could also relates to other co-factors, such as IGF-1 [24].

Historically, acromegaly has been considered a risk factor for secondary neoplasms, but this theory has been challenged, as carcinogenesis mechanism(s) are more complex clinically [25]. There are no studies comparing the risk of cancer between patients with acromegaly in adulthood and in childhood.

There are also limitations to clinical studies such as study design (mainly retrospective) and patient cohort characteristics age, therapeutic strategies, disease-independent environmental risk factors, and diagnostic delay. Also, studies with higher cohort numbers have not considered family and genetic history and exposure to particular risk factors. National registries on average contain more complete epidemiology data, disease control information, and therapeutic strategies, with perhaps less comorbidity information [26]. Despite some data limitations, change in the causes of mortality in patients with acromegaly has been observed over the last three decades, with a shift from cardiovascular diseases as the leading cause of death to neoplastic causes [14]. Additionally, mortality risks reportedly vary from country to country with sex differences noted [7, 27]. Screening for acromegaly complications is highly debated and there are no universal guidelines available to define methods and timing [28–30]. It is therefore difficult to fully understand and identify the most appropriate strategy for cancer screening in patients with acromegaly.

## Methods

All studies concerning cancer and screening in patients with acromegaly over the past 50 years were searched and selected in the PubMed database (years 1973–2024). Concerning screening in the general population, the protocols of the different societies dealing with these diseases were searched. For each neoplasm, we then compared the screening methods and periods inherent in the general population and patients with acromegaly. Our narrative review focuses on studies reporting screening programs and risk factors for cancer. The literature in PubMed database on neoplasms in patients with acromegaly in the last five decades was reviewed. Searched terms included “acromegaly”, “neoplasms in acromegaly”, “secondary tumors”, “cancer screening”, “colon neoplasm screening”, “breast neoplasm screening”, “prostate neoplasm screening”, “thyroid neoplasm screening”, “cancer risk factors”, “pathogenetic mechanism of tumors in acromegaly”, “secondary tumor management”, “disease control”, “medical therapy acromegaly”, “serum GH levels”. Further references found in the articles mentioned above were also searched further if relevant to the topic.

## Discussion

Colorectal, thyroid, lung, prostate, breast, and stomach cancers are those most commonly found in patients with acromegaly [31], thus we have reviewed here these neoplasms which currently are screened for in screening protocols.

### Risks factors for cancer in patients with acromegaly

As life expectancy of the general population has increased, obesity too, has also increased, particularly as related to certain malignancy risk [32]. Over the past two decades, the relationship between obesity and cancer has been widely investigated [33–35].

A significant association between obesity and colon, thyroid, breast, and prostate cancer(s) has been described [36–39]. In patients with acromegaly, an increase in body weight occurs typically after pituitary surgery, particularly an increase in visceral fat [40–42]. Increased visceral fat was also observed during therapy with pegvisomant [43, 44]. Growth hormone inhibits activity of the lipoprotein lipase in adipose tissue, increasing the efflux of free fatty acids to the liver, and therefore promoting the insulin resistance, increased synthesis of triglycerides, and reduced high-density lipoprotein (HDL) levels, and body fat [45]. This complex metabolic pathway may play a crucial role in determining an increased risk for secondary neoplasm in patients with acromegaly.

The overreplacement of glucocorticoid replacement in patients with hypopituitarism could also theoretically play a role as there is an increased cancer risk in patients with Cushing syndrome [46]. Adrenal insufficiency (AI) in patients with acromegaly who are undergoing transsphenoidal surgery is a very important complication and occurs postoperatively in 3% to 18% of patients [47, 48]. Unfortunately, patients with AI are often exposed to higher levels of glucocorticoids than the general population [49], and after prolonged periods of exposure, this can lead to complications such as diabetes mellitus, hypertension, dyslipidemia, and bone fragility fractures [50–52]. The immuno-suppressive and anti-inflammatory effects of glucocorticoids are used for the treatment of several diseases [53, 54]. However, the immune system plays an important role in preventing the development and progression of neoplasms, also in somatotroph tumors [55–59]. Chronic exposure to glucocorticoids may down-regulate the immune response against cancer, potentially playing a role in tumor escape [60, 61]. To date, there is no conclusive evidence on the risk of second neoplasia(s) development in patients who are taking glucocorticoid replacement therapy and have secondary AI but further studies are needed based on new data on CS.

Diabetes mellitus is a prevalent disease, one that significantly impacts individual(s) health, globally [62, 63]. Several studies have reported etiopathogenetic mechanisms that expose patients with diabetes mellitus to an increased risk of neoplasms [64, 65], including hyperinsulinemia, considered a key risk factor [66]. Patients with diabetes mellitus appear to have a higher mortality rate than subjects without diabetes [67, 68]. For patients with acromegaly, diabetes mellitus is more common than in the general population [31, 69–71] and prevalence increases with disease duration [72]. Biochemical control of acromegaly reduces the risk of developing new-onset diabetes [73]. Acromegaly predisposes to insulin resistance, and the diabetogenic effect of GH would seem to outweigh the insulin-sensitizing effect of IGF-1 [74]. Therefore, patients with acromegaly who are naïve to treatments have a higher homeostatic model assessment for insulin resistance (HOMA-IR) than the general population [75]. Additionally, insulin resistance improves after transsphenoidal surgery, correlating with serum IGF-1 levels before and after surgery [76]. Therefore, it is necessary to take diabetes mellitus into account when assessing the neoplastic risk of a patient with acromegaly.

We have reviewed all the studies available in the literature indicating the number of neoplasms found in different patient groups and reported the most frequent ones ( Table 1) [20, 21, 23, 77–83]. The difficulty in statistical comparison between data from different studies is due to several variables including different periods of patient observation, different treatment strategies used, and individual risk factors unrelated to acromegaly. In addition, some types of data (for example, mean age at diagnosis of acromegaly) is not available for all case series.

**Table 1:** Number of neoplasms found in different patient groups and reported the most frequently diagnosed. References [20, 21, 23, 77–83]

Author and period of observation	N. of patients	Sex (% of male)	Country	Mean age at diagnosis of acromegaly	N. of cancers	Most frequent cancers
Freda et al. (2024) [20] 1996 – 2019	598	52	New York	-	156	35 Breast 22 Thyroid 18 Prostate 13 Colon 12 Haematological
Dal J et al. (2018) [77] 1978 – 2010	529	51	Denmark	47.4	81	10 Colorectal 9 Breast 9 Prostate 5 Urinary tract 5 Haematological
Terzolo et al. (2017) [78] 1980 – 2002	1512	41	Italy	45.0	124	22 Breast 20 Colorectal 13 Thyroid 10 Kidney
Maione et al. (2017) [23] −2012	926	46	France	43 for male 48.5 for female	94	21 Breast 18 Thyroid 15 Colorectal 9 Prostate 8 Haematological
Wolinski et al. (2017) [79] 2005 – 2016	200	36	Poland	47.6	29	14 Thyroid 7 Breast 4 Colon
Petroff et al. (2015) [21]	445	45	Germany	45.7	46	16 Breast 4 Colorectal 3 Thyroid 2 Lung
Kauppinen et al. (2010) [80] Diagnosis of acromegaly 1980 – 1999	331	-	Finland	-	48	6 Colorectal 6 Thyroid 6 Breast 5 Urinary tract 5 Haematological
Kurimoto et al. (2008) [81]	140	39	Japan	-	22	10 Colorectal 5 Thyroid 4 Breast 2 Stomach
Baris et al. (2002) [82] 1965 – 1993	1634	46	Denmark and Sweden	50.7	117	36 Colorectal 23 Haematological 20 Breast 14 Lung 13 Prostate
Orme et al. (1998) [83] 1958 – 1995	1239	-	United Kingdom	-	79	16 Colorectal 14 Breast 6 Bronchus

No significant data are available in the literature linking therapy given for acromegaly and the risk of cancer.

### Colorectal cancer

Colorectal cancer is the third most frequently diagnosed neoplasm and accounts for 10% of all new cancer cases, globally (1.9 million cancer cases per year) [84, 85]. An association between colorectal cancer and acromegaly has been investigated, and IGF-1 and GH excess are clearly implicated in the mechanisms of colon carcinogenesis [86–91]. In a case–control study, it was observed that serum IGF-1 values were significantly higher in patients with colorectal cancer, than in subjects without colorectal cancer [92]. A meta-analysis (nine studies) showed that the risk of developing colorectal neoplasia in patients with acromegaly is higher than in the general population [93]. The likelihood of developing adenomatous polyps is also higher in patients with acromegaly, particularly in young males [94]. The process of neoplastic transformation of adenomatous lesion(s) to colorectal cancer is now strongly recognized [95]. In one study, serum GH levels were correlated directly with finding polyps during a colonoscopy, particularly adenomatous polyps, and serum IGF-1 levels also correlated directly with the likelihood of finding adenomatous and hyperplastic polyps [96]. Furthermore, patients with acromegaly have an increased risk of transformation of an adenomatous polyp to a neoplasm of the colon-rectum [97].

Interestingly, one study showed that the size of the GH-secreting adenoma was an independent risk factor for development per se of polyps [98]. One possible explanation for this phenomenon is the positive correlation between tumor volume and increased GH secretion [99]. An additional risk factor for colorectal neoplasia in patients with acromegaly is an abnormally long large intestine (dolichocolon) [100]; 15–20% longer than the general population [101]. Consequently, there is a higher stochastic risk due to excess tissue that could potentially evolve into neoplastic tissue [101]. Despite these risk factors, and some selection bias a meta-analysis showed that the overall risk of developing neoplasms is only slightly increased in patients with acromegaly [77]. However, in an Italian study up to 19.3% of patients with acromegaly, under 40 years of age, also had a colorectal neoplasm at the time of acromegaly diagnosis vs 4.4% of controls [102].

Screening and follow-up for colorectal cancer in patients with acromegaly remains highly debated. Of note, it is recommended that a colonoscopy include a complete colon inspection, including the cecum since in patients with acromegaly most lesions are located in the ascending colon [103]. Colonoscopy timing and follow up details in patients with acromegaly, active disease or in remission, are yet to be elucidated. Studies on the outcome of at least two colonoscopies in the same groups of patients with acromegaly reveal a lower frequency of new colorectal cancer at a second colonoscopy (Table 2) [96, 102, 104, 105]. Colonoscopies were conducted at least 2 years following the first colonoscopy, reflecting population cohorts characterized by biochemical remission [96, 102, 104, 105]. No other risk factors for colorectal neoplasia have been identified. Additional data is required to more accurately determine the incidence of colorectal neoplasia in patients with acromegaly, while also taking into account the updated diagnosis criteria [12] and improved biochemical control over time [23, 41]. National registries (so far) have not reported the number of and time intervals between colonoscopies performed to screen colorectal cancer. This information could help to determine appropriate colonoscopy timing in patients with acromegaly. For now, and according to the British Society of Gastroenterology (BSG), a colonoscopy should be performed after 40 years of age. The Pituitary Society, the Endocrine Society, and the Acromegaly Consensus Group (ACG) indicate that a first colonoscopy examination should be at the time of diagnosis of acromegaly, regardless of the patient's age [106]. During follow-up, the BSG, Pituitary Society, and Endocrine Society recommended repeating a colonoscopy after 10 years, in acromegaly patients with normal baseline colonoscopy. In patients with previous findings of colonic polyps (adenoma), and in patients the active acromegaly, the BSG recommends repeating a colonoscopy after 3 years, the Endocrine Society after 5 years and the ACG recommends that further screening should be based on initial findings and gastroenterology specialists.

**Table 2:**
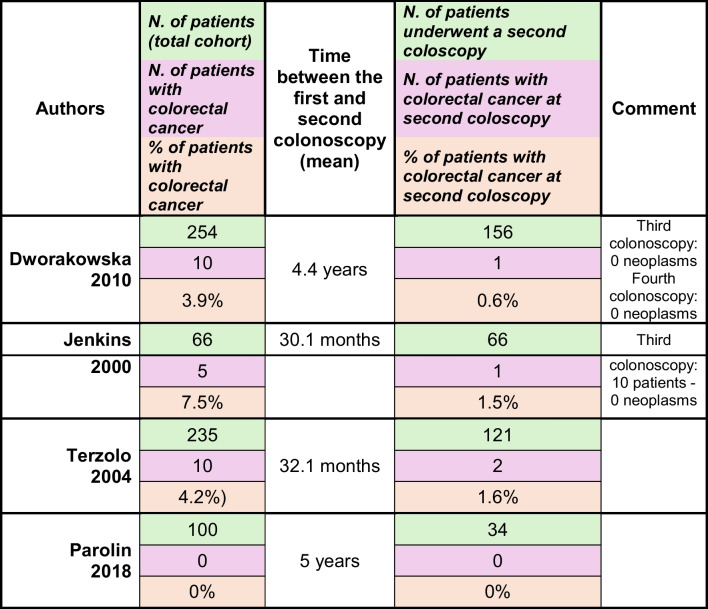
Frequency of colorectal cancer according to number sequence and time of colonoscopy

**Fig. 1:**
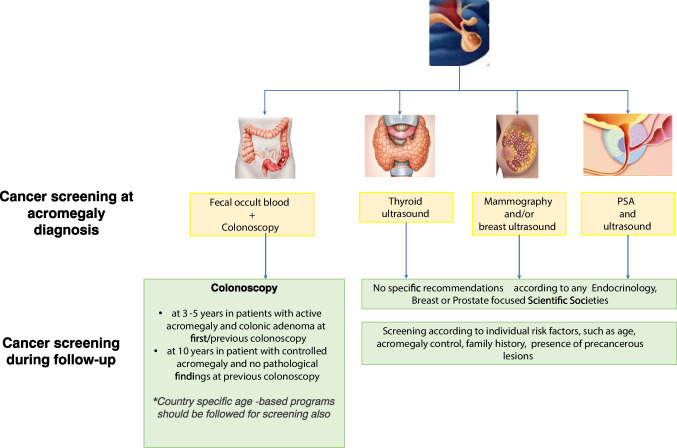
Proposed flow-chart for screening of colon, thyroid, breast and prostate cancer at acromegaly diagnosis and during follow-up

No guidelines are available regarding use of fecal occult blood screening. In a study by Bogazzi et al., this method was inferior in detection of colorectal neoplasms in patients with acromegaly compared to colonoscopy [107]. However, as a faster and less expensive method, it could be considered as screening tool, with timing dependent on disease control and individual risk factors, in between colonoscopies. A suggested flow-chart for screening and follow-up of colon cancer in patients with acromegaly *as per* available literature is provided (Fig. 1.). It remains essential to establish an appropriate and individualized patient screening and follow-up program with regards to disease activity.

### Thyroid cancer

In the general population, over the past four decades, the incidence of thyroid cancer has progressively increased to 14 cases/100,000 [108]. This is most likely due to increased numbers of imaging techniques performed, especially ultrasound [109, 110]. The most frequent histological type is papillary thyroid carcinoma [111], with a mortality rate of 1–2% at 10 years [112].

According to available epidemiology data, the incidence of thyroid carcinoma in patients with acromegaly is not on the increase [14, 113]. However, from a pathogenic point of view, IGF-1 directly acts on thyroid carcinogenesis. In 1989, Yashiro et al. demonstrated the presence of IGF-1 receptors on human thyroid cells [114]. In a study conducted in children and adolescents, aggressive behavior was identified in thyroid tumors expressing the IGF-1 receptor [39]. Pidchenko et al. reported increased IGF-1 and IGF-2 serum levels in patients with papillary thyroid carcinoma without acromegaly, compared to the healthy population [115]. Keskin et al. reported a more intense expression of galectine-3 and IGF-1 in thyroid tumors of patients with acromegaly than in thyroid tumors of patients without GH excess [116]. A low prevalence of the BRAF V600E mutation was reported in patients with acromegaly with thyroid neoplasia, which suggests that this mechanism would appear not to be the main cause of the development of thyroid tumors in patients with acromegaly [117]. Overall, studies seem to suggest a mechanism of carcinogenesis more closely linked to the hypersecretion of and elevated circulating levels of IGF-1.

However, the size of thyroid nodules does correlate with acromegaly disease activity; smaller nodules were reported in acromegaly patients with disease controlled during treatment with SRLs [118]. Thyroid size also has been shown to correlate with disease activity [119]. Furthermore, thyroid nodules have greater stiffness, as determined by elastosonography, in the active phase of acromegaly disease. These characteristics do not seem to correlate with nodule malignancy [120].

The link between high incidence of thyroid tumors in patients with acromegaly in comparison to general population is inconclusive [121]. There could be various different risk factors (e.g. radiation), and the number of screening tests performed in the population.

In a study that was conducted in 60 patients with acromegaly who all underwent thyroid ultrasound, 75% had thyroid nodules. Papillary thyroid carcinoma (PTC) occurred in 25% of the patients, who had significantly higher levels of IGF-1 [122]. Of note, the risk of PTC is increasing worldwide in patients who undergo ultrasound [23, 110] and clinical relevance remains unknown. Rogozinski et al. showed an increased percentage of high-risk cytological thyroid nodules in patients with acromegaly compared to patients without GH excess (25% vs. 9%) [124]. Furthermore, same authors recommend screening for thyroid morphology in patients with acromegaly by ultrasound and if necessary fine-needle aspiration (FNA) [125, 126].

A study comparing clinical outcomes in patients from two large referrals centers in two continents (Europe and US) found that patients who undergo thyroid ultrasound screening in clinic have more thyroid nodules than those who undergo thyroid ultrasound due to the presence of palpable nodules. Different rates of iodine deficiency could also play a role in the study results [127]. To date, there are no guidelines recommending ultrasound thyroid screening for patients with acromegaly, and the ATA guidelines from 2015 do not include acromegaly as a risk factor for developing thyroid carcinoma [128].

The Pituitary Society guidelines from 2020 recommend a thyroid ultrasound in patients with acromegaly only if palpable nodules are found on clinical examination [129]. Similarly, the ACG Consensus notes that ultrasound screening in all patients is not needed, but thyroid ultrasound and careful evaluation is recommended in those with palpable thyroid nodules and other risk factors for thyroid cancer; this is consistent with guideline recommendations for the general population. It is not clear whether it is necessary to perform a thyroid ultrasound in all patients with acromegaly who are in areas of higher risk of thyroid nodules (e.g. iodine deficiency, radiation) and if yes, what timing is most appropriate (Fig. 1).

### Breast cancer

In the general population, breast cancer is overall the most diagnosed malignancy in the world with an incidence of 48 cases/100,000 [130], this has progressively increased over the last 25 years [131].

To date and worldwide, in the general population, mammography is the most widely used screening tool for early detection of breast cancer in asymptomatic patients [132]. Breast ultrasound is also used in some women with dense breast, but usually after a positive mammogram. Regular screening for breast neoplasia can reduce mortality in asymptomatic patients [133]. Various breast cancer society(s) recommend screening, at different ages for a first mammography with subsequent differing frequencies. The American Cancer Society and the American Society of Clinical Oncology, in 2015, recommended an annual mammogram in women between the ages of 45 and 54 years, and every year or every 2 years in women older than 54 years [134]. The most recent guidelines from the US Task Force recommends that all women get screened for breast cancer every other year, starting at age 40 years and continuing through age 74 years, to reduce their risk of dying from this disease [135].The European Commission Initiative on Breast Cancer, in 2019, suggested a mammography every 2 years for patients aged between 45 and 69 years [136]. The National Health Service Breast Screening Program of the United Kingdom, in 2016, recommended mammography screening every 2 years in women aged between 50 and 70 years [137].

Acromegaly is not included as a risk factor for the development of breast neoplasia by any of the noted above society’s guidelines. To date, there is no consensus on the method and timing of breast cancer screening in patients with acromegaly, and it is still debated whether acromegaly is a risk factor for the development of breast cancer. One study shows a fourfold increased risk for breast cancer in female patients with acromegaly [138]. Murphy et al., showed a correlation between the increased risk of breast neoplasia in premenopausal and postmenopausal patients and elevated serum concentrations of IGF-1 [139]. Orme et al., reported a 2.9-fold increased mortality risk in patients with acromegaly and serum GH levels > 10 ng/mL [83]. Tagliafico et al., demonstrated a positive correlation between the serum IGF-1 levels and the breast density in patients with acromegaly [140]. However, the increased density of breast tissue, which is frequent in patients with acromegaly [141], is considered an independent risk factor for breast cancer [142], also because it reduces the sensitivity of mammography in identifying small lesions [143, 144].

The European Society of Breast Imaging (EUSOBI) recommended, in 2022, to perform a breast magnetic resonance imaging (MRI) every 2 to 3 years, in females aged from 50 to 70 years with increased breast density [145].

Although several studies have reported a correlation between acromegaly and breast neoplasia [19, 77, 146, 147], there are currently no guidelines for breast cancer screening in patients with acromegaly.

The average age at diagnosis of acromegaly is 45.2 years, and although up to 5% of cases may be diagnosed before age 20 years, the most common range is between 40 and 50 years [26, 148]. Many women would be at the age of screening per normal population, which is now 40 years of age. However, it is possible that women with other risk factors including long term uncontrolled acromegaly might benefit of early screening. Therefore, additional data should be collected to establish the most appropriate breast cancer screening pathway for patients with acromegaly (Fig. 1).

### Prostate cancer

Prostate cancer is one of the most frequent malignancies in males worldwide, with a current age-related incidence of around 156 cases/100,000 [149]. Screening in the general population is still debated in the medical community due to risks of overdiagnosis and increased unnecessary medical procedures. Serum prostate-specific antigen (PSA) testing is the most discussed method in terms of cost-effectiveness and ease of use [150, 151]. A few clinical case reports have described increased prostate neoplasia in patients with acromegaly [77, 81, 152, 153], but an association between prostate cancer and acromegaly is also strongly debated. However, there are no studies of adequate sample size that significantly support an increased risk of prostate neoplasia.

High serum levels of IGF-1 and GH could lead to increased prostate volume and structural rearrangement with the development of calcifications, nodular and cystic lesions [154]. A high frequency of urinary symptoms in patients with acromegaly younger than 40 years of age and a high frequency of prostate structural alterations in those older than 40 years of age has been reported [155]. Benign prostatic hyperplasia would appear to be more frequent in patients with acromegaly than in the general population [154]. Treatment with octreotide induced a reduction of prostate volume [156], with no clear evidence of pathogenetic mechanisms. Given the unclear association between increased IGF-1 serum levels and prostate neoplasia and the strong need for further data, we consider it necessary to monitor morphological changes in the prostate of patients with acromegaly. Screening for prostate cancer should be conducted according to baseline examination findings and individual risk factors, including treatment with testosterone replacement for central hypogonadism (Fig. 1).

## Conclusions

Acromegaly-related mortality, historically, was mainly due to cardiovascular and respiratory comorbidities. However, in recent years, the incidence of secondary neoplasia(s) in patients with acromegaly has increased, because of higher rates of biochemical control and a longer life expectancy. Due to the rarity of acromegaly, a limited number of studies, small sample size, and the different reported study screening procedures, no conclusive evidence is available to define the most efficacious screening protocol for neoplasms. We suggest that screening be personalized according to individual risk factors, including the duration of uncontrolled acromegaly. We advocate for prospective, large cohort studies to validate epidemiology data, acromegaly-related risk factors, and screening protocols for various neoplasia(s) that are customized and individualized for patients with acromegaly. Data collection conducted through cohort studies and registry should also include individual and environmental risk factors, familiarity for neoplasms screening, comorbidities, and therapeutic strategies used to control acromegaly.

## Data Availability

No datasets were generated or analysed during the current study.

## Abbreviations

ACG: Acromegaly Consensus Group
AKT: Protein kinase-B
ATA: American Thyroid Association
BSG: British Society of Gastroenterology
ECIBC: European Commission Initiative on Breast Cancer
EUSOBI: European Society of Breast Imaging
FNA: Fine-needle aspiration
Gal-3: Galectine-3
GH: Growth hormone
IGF-1: Insulin growth hormone-1
IPSS: International Prostatic Symptoms Score
MAPK: Mitogen-activated protein kinase
NHSBSP: National Health Service Breast Screening Program
PI3K: Phosphatidylinositol 3-kinase
PSA: Prostate-specific antigen
SMR: Standard mortality ratio
STAT5: Signal transducer and activator of transcription 5
